# The art of life and death: 14 year follow-up analyses of associations between arts engagement and mortality in the English Longitudinal Study of Ageing

**DOI:** 10.1136/bmj.l6377

**Published:** 2019-12-18

**Authors:** Daisy Fancourt, Andrew Steptoe

**Affiliations:** 1 Department of Behavioural Science and Health, University College London, London WC1E 7HB, UK

## Abstract

**Objective:**

To explore associations between different frequencies of arts engagement and mortality over a 14 year follow-up period.

**Design:**

Prospective cohort study.

**Participants:**

English Longitudinal Study of Ageing cohort of 6710 community dwelling adults aged 50 years and older (53.6% women, average age 65.9 years, standard deviation 9.4) who provided baseline data in 2004-05.

**Intervention:**

Self reported receptive arts engagement (going to museums, art galleries, exhibitions, the theatre, concerts, or the opera).

**Measurement:**

Mortality measured through data linkage to the National Health Service central register.

**Results:**

People who engaged with receptive arts activities on an infrequent basis (once or twice a year) had a 14% lower risk of dying at any point during the follow-up (809/3042 deaths, hazard ratio 0.86, 95% confidence interval 0.77 to 0.96) compared with those who never engaged (837/1762 deaths). People who engaged with receptive arts activities on a frequent basis (every few months or more) had a 31% lower risk of dying (355/1906 deaths, 0.69, 0.59 to 0.80), independent of demographic, socioeconomic, health related, behavioural, and social factors. Results were robust to a range of sensitivity analyses with no evidence of moderation by sex, socioeconomic status, or social factors. This study was observational and so causality cannot be assumed.

**Conclusions:**

Receptive arts engagement could have a protective association with longevity in older adults. This association might be partly explained by differences in cognition, mental health, and physical activity among those who do and do not engage in the arts, but remains even when the model is adjusted for these factors.

## Introduction

Interest in the salutogenic (health promoting) benefits of the arts is increasing. Arts activities are classified as “multimodal” health interventions because they combine multiple psychological, physical, social, and behavioural factors with an intrinsic aesthetic motivation to engage.^1^ While previous studies have shown the association between arts engagement and the prevention and treatment of mental and physical health conditions, including depression, dementia, chronic pain, and frailty,^2^ ^3^ ^4^ whether arts engagement actually confers survival benefits remains unclear. Some research has proposed that the universality of art and the strong emotional responses it induces are indications of its association with evolutionary adaptations,^5^ ^6^ while other research has questioned whether art is an evolutionary parasite, with no particular evolutionary benefits to our species.^7^

Within health research, arts engagement could be linked to longevity by alleviating chronic stress and depression, and providing emotional, cognitive, and social coping resources that support biological regulatory systems and behavioural choices.^8^ Arts engagement is also known to enhance social capital, which builds individual and collective resources,^9^ ^10^ and to reduce loneliness, which is associated with mortality.^11^ Arts engagement can support cognitive reserve,^2^ ^12^ and promotes empathy, social perception, and emotional intelligence, which are all linked to a greater chance of survival.^13^ Arts engagement could help to reduce sedentary behaviours, which are well established predictors of cardiovascular health and immune function,^14^ ^15^ and might also reduce risk taking behaviours. Arts engagement is linked to a greater sense of purpose in life, which is itself associated with better immune function and healthier behaviours.^16^ Further, creativity and imagination, which are an intrinsic part of artistic engagement, have been linked to increased chance of survival across the evolution of our species.^17^ So there is a strong theoretical rationale that underlies the hypothesis that arts engagement could be linked to people’s chance of survival.

In further support of this hypothesis, studies that focus more broadly on “leisure” (active and receptive arts activities alongside a heterogeneous group of other activities, including studying, eating out, gardening, having a hobby, and religious attendance) have found protective associations with premature mortality.^18^ ^19^ ^20^ ^21^ ^22^ Additionally, two Scandinavian studies of receptive arts engagement (especially going to the cinema, concerts, art exhibitions, and museums) have also found preliminary evidence of protective benefits from attending cultural events.^23^ ^24^ However, these previous studies have drawn exclusively on Scandinavian datasets with baseline data collected in the 1970s, 1980s, or 1990s.^18^ ^19^ ^20^ ^21^ ^23^ ^24^ Given that arts engagement has different values and patterns across generations and countries, we lack evidence from the current generation of older adults in other countries. Further, these previous studies have omitted important confounding variables and paid little attention to the frequency of engagement required for associations with longevity to be seen.

In this study we explored the association between different frequencies of arts engagement and mortality in adults in England aged 50 years and older. The follow-up period was 14 years. We considered a comprehensive list of covariates, including demographics, socioeconomic status, health conditions, behaviours, cognitive state, and other social and civic engagement.

## Methods

### Participants

We included participants from the English Longitudinal Study of Ageing (ELSA), a multiwave, nationally representative cohort study of community dwelling adults aged 50 years and older. Households included had previously responded to the Health Survey for England in 1998, 1999, or 2001 (wave 0).^25^ ELSA started in 2002 and the same people have been interviewed every two years. For our analysis, we used data from wave 2 of ELSA (2004-05) as our baseline because this wave contained relevant data on our exposure and covariates. Core participants who provided data at wave 2 were included in our study and followed up by linkage to mortality data from the National Health Service central register. The latest available mortality data were from March 2018 (an average follow-up of 12 years and two months, maximum 13.8 years).

**Fig 1:**
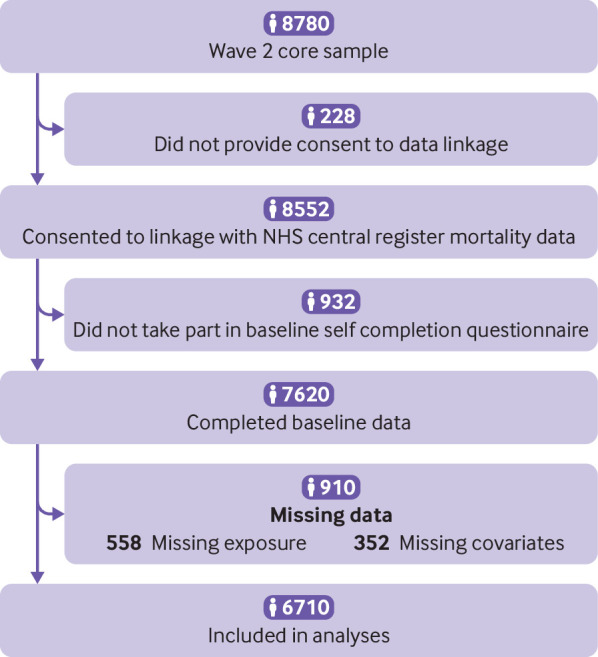
Flowchart of participants included in the study

Wave 2 assessed 8780 core participants. Our inclusion criteria were consent to data linkage and availability of linked data, and completion of baseline self completion interview and questionnaire, which provided data on baseline receptive arts engagement and covariates. A total of 8552 participants gave consent to data linkage and their records were followed up (97.4%); 7620 completed the questionnaire; and 6710 provided full usable data across all measures so were included in analyses (fig 1).

### Measures

We focused specifically on receptive arts activities at baseline (2004-05), including going to the theatre, concerts, opera, museums, art galleries, and exhibitions (“receptive arts engagement”).^26^ Frequency of engagement with any of these activities was categorised as never, infrequent (less than once a year; once or twice a year), or frequent (every few months; monthly or more). 

We identified factors that predicted receptive arts engagement and mortality by using directed acyclic graphs and included these factors as covariates.^27^ Demographic and socioeconomic covariates were age, sex, marital status (married or cohabiting *v* single, widowed, or divorced), and ethnicity (white British *v* other); educational qualifications (no educational qualifications; education to GCE, O level, national vocational qualification (NVQ) 2 (qualifications at age 16); education to NVQ3, GCE, A level (qualifications at age 18); higher qualification, NVQ4, NVQ5, degree); total non-pension wealth (which combines net financial and physical wealth with net owner occupied housing wealth, categorised in fifths)^28^; employment status (working full or part time *v* not working or retired); and occupational status (managerial and professional occupations, intermediate occupations, small employers and self employed, lower supervisory and technical occupations, and semiroutine occupations; categorised using the five item National Statistics Socio-economic Classification).

Health related covariates were self reported eyesight (fair or poor *v* excellent, very good, or good), self reported hearing (fair or poor *v* excellent, very good, or good), and depressive symptoms (as a continuous measure using the Centre for Epidemiological Studies Depression scale); whether participants had been diagnosed as having any other psychiatric condition (including anxiety, psychosis, major depression, mood swings, or other emotional problems); whether participants reported currently having a diagnosis of cancer, lung disease, or cardiovascular disease (including high blood pressure, angina, a previous heart attack, heart failure, a heart murmur, an abnormal rhythm, diabetes, a previous stroke, high cholesterol, or other heart trouble); whether participants reported having a history of any other long term condition (including arthritis, asthma, osteoporosis, Parkinson’s disease, Alzheimer’s disease, or dementia); whether participants currently smoked; frequency of alcohol consumption (1-2 days a week, 3-4 days a week, 5-6 days a week, or daily); whether participants were sedentary (categorised as engaging in mild, moderate, or vigorous activity less than once a week); whether participants had any problems with their mobility (including walking 100 yards, sitting for two hours, getting up from a chair, climbing stairs, stooping, extending their arms, moving large objects, carrying weights, or picking up objects); whether participants had any difficulty in carrying out daily tasks (including dressing, bathing, eating, using a toilet, shopping, taking drugs, or making telephone calls); whether participants had osteoporosis; and cognition (an average of standardised scores of memory, executive function, processing speed, and orientation in time using validated measures from a neuropsychological battery^29^).

Social covariates included perceived loneliness (measured using the four item University of California, Los Angeles (UCLA) loneliness scale); the number of reported close friends (0, 1-2, 3-5, and 6 or more); whether participants lived alone; the frequency with which participants engaged in civic activities (including political parties, trade unions, environmental groups, tenants or residents associations, neighbourhood watch, church or religious groups, charitable associations, evening classes, social clubs, sports clubs, exercise classes, or other clubs or societies); the frequency with which people saw friends, family, or children (less than once a month, once or twice a month, once or twice a week, or three or more times a week); and whether participants had a hobby or pastime.

### Statistical analysis

Table 1 shows the importance of baseline differences between participants based on end mortality status and arts engagement, which was calculated using χ^2^ tests. We estimated cumulative mortality by using the Kaplan-Meier method. Unadjusted and adjusted hazard ratios of mortality and 95% confidence intervals were calculated using Cox proportional hazards regression models. We measured survival time in months from birth date to death, censoring (the date of the last interview before drop out), or latest available follow-up (165 months from baseline). Sensitivity analyses that used survival time from baseline interview produced comparable results. We adjusted models for demographic variables (age, sex, marital status, ethnicity, educational qualifications, wealth, employment status, and occupational status); health related variables (eyesight, hearing, depressive symptoms, other psychiatric conditions, diagnosis of cancer, lung disease or cardiovascular disease, history of any other long-term condition, smoking, alcohol consumption, sedentary behaviours, mobility, problems in undertaking activities of daily living, osteoporosis, and cognition); and social covariates (loneliness, number of close friends, living alone, frequency of civic engagement, frequency of social engagement, and whether participants had a hobby or pastime).

**Table 1:** Demographic characteristics of sample according to deaths and receptive arts engagement

Variable	No of deaths (%)	P	Engagement (%)			P
			Never	Infrequently	Frequently	
**Receptive arts engagement**						
Never	837/1762 (47.5)	<0.001	—	—	—	—
Infrequently	809/3042 (26.6)	—	—	—	—	—
Frequently	355/1906 (18.6)	—	—	—	—	—
**Demographics**						
Sex:						
Men	1060/3115 (34)	<0.001	28.4	45.3	26.4	<0.001
Women	941/3595 (26.2)	—	24.4	45.4	30.2	—
Age:						
52-59	178/2127 (8.4)	<0.001	19.5	49	31.5	<0.001
60-69	459/2353 (19.5)	—	22.8	46.1	31.1	—
70-79	796/1592 (50)	—	31.7	43.1	25.2	—
≥80	568/638 (89)	—	47.8	35.7	16.5	—
Marital status:						
Single	849/1933 (43.9)	<0.001	34.6	39.2	26.2	<0.001
Married/coupled	1152/4777 (24.1)	—	22.9	47.8	29.3	—
**Socioeconomic status**						
Education:						
Degree	189/894 (21.1)	<0.001	5.2	35.7	59.2	<0.001
Qualifications to age 18	517/1970 (26.2)	—	18	50.4	31.7	—
Qualifications to age 16	239/1207 (19.8)	—	15.7	51.7	32.6	—
No qualifications	1056/2639 (40)	—	44.4	42	13.6	—
Employment:						
Not currently working	1781/4394 (40.5)	<0.001	31.4	43.2	25.4	<0.001
Working full or part time	220/2316 (9.5)	—	16.6	49.4	34	—
Wealth:						
Lowest fifth (median £1833)	521/1120 (46.5)	<0.001	54.7	33.7	11.6	<0.001
2nd fifth (median £112 000)	410/1281 (32)	—	35.1	46	19	—
3rd fifth (median £188 399)	403/1400 (28.8)	—	24	51.1	24.9	—
4th fifth (median £285 000)	369/1439 (25.6)	—	15.8	50.1	34.1	—
Highest fifth (median £522 500)	298/1470 (20.3)	—	9.3	43.5	47.3	—
Occupational status:						
Professional or managerial	588/2225 (26.4)	<0.001	12.9	44.3	42.9	<0.001
Intermediate	472/1701 (27.8)	—	22.1	49.2	28.8	—
Routine or manual	941/2784 (33.8)	—	39.6	43.8	16.6	—
**Health**						
Depressive symptoms:						
CES-D score 0-2	1445/5307 (27.2)	<0.001	22.5	47	30.5	<0.001
CES-D score 3-8	556/1403 (39.6)	—	40.4	39.2	20.4	—
Other psychiatric condition:						
No	1995/6467 (30.2)	<0.001	26	45.5	28.5	0.026
Yes	46/243 (18.9)	—	33.7	40.3	25.9	—
Eyesight:						
Poor or fair	70/124 (56.5)	<0.001	52.4	34.7	12.9	<0.001
Good, very good, or excellent	1931/6586 (29.3)	—	25.8	45.5	28.7	—
Hearing:						
Poor or fair	144/306 (47.1)	<0.001	45.4	41.8	12.8	<0.001
Good, very good, or excellent	1857/6404 (29)	—	25.3	45.5	29.2	—
Diagnosis of cancer:						
No	1919/6560 (29.3)	<0.001	26.3	45.3	28.4	0.97
Yes	82/150 (54.7)	—	25.3	46	28.7	—
Diagnosis of lung disease:						
No	1944/6605 (29.4)	<0.001	25.9	45.5	28.6	<0.001
Yes	57/105 (54.3)	—	48.6	38.1	13.3	—
Diagnosis of cardiovascular disease:						
No	1106/4196 (26.4)	<0.001	24	45.8	25.3	<0.001
Yes	895/2514 (35.6)	—	30.1	44.6	30.3	—
Diagnosis of other long term condition:						
No	1747/5937 (29.4)	0.05	25.9	45.4	28.7	0.11
Yes	254/773 (32.9)	—	29	45.2	25.9	—
Cognition:						
Lowest quarter	915/1678 (54.5)	<0.001	44.6	40.2	15.2	<0.001
2nd quarter	513/1681 (30.5)	—	28.4	47.3	24.3	—
3rd quarter	350/1674 (20.9)	—	20	46.8	33.2	—
Highest quarter	223/1677 (13.3)	—	12	47.1	41	—
Problems with mobility:						
No	1485/3861 (38.5)	<0.001	32.1	45	22.9	<0.001
Yes	516/2849 (18.1)	—	18.4	45.8	35.9	—
Impairment in daily living tasks:						
No	1143/4904 (23.3)	<0.001	20.3	47.5	32.3	<0.001
Yes	858/1806 (47.5)	—	42.6	39.6	17.8	—
Osteoporosis:						
No	1886/6453 (29.2)	<0.001	26	45.6	28.4	0.028
Yes	115/257 (44.8)	—	33.1	38.9	28	—
**Health behaviours**						
Sedentary lifestyle:						
No	1727/6276 (27.5)	<0.001	24.2	46.2	29.6	<0.001
Yes	274/434 (63.1)	—	56.2	32.3	11.5	—
Alcohol consumption:						
Less than once a week	928/2612 (35.5)	<0.001	35.8	43.1	21.1	<0.001
1-2 times a week	460/1692 (27.2)	—	22.9	50.4	26.7	—
3-4 times a week	175/806 (21.7)	—	16.1	49.4	34.5	—
≥5 times a week	438/1600 (27.4)	—	19.4	41.6	39.1	—
Current smoker:						
No	1654/5759 (28.7)	<0.001	23.5	46.2	30.4	<0.001
Yes	347/951 (36.5)	—	43.2	40.3	16.5	—
**Social factors**						
No of close friends:						
0	438/1136 (38.6)	<0.001	38.9	43.1	18.1	<0.001
1-2	663/2174 (30.5)	—	29.2	47.1	23.7	—
3-5	594/2246 (26.5)	—	20.9	45.2	33.8	—
≥6	306/1154 (26.5)	—	18.6	44.5	36.9	—
Loneliness score:						
Lowest quarter	473/2181 (21.7)	<0.001	16.5	47.2	36.3	<0.001
2nd quarter	366/1264 (30.2)	—	23.7	48.4	27.9	—
3rd quarter	672/2057 (24.1)	—	29.9	44.2	25.9	—
Highest quarter	490/1208 (46.5)	—	40.6	40.6	18.9	—
Live alone:						
No	1244/5085 (24.5)	<0.001	23.8	47.3	28.9	<0.001
Yes	757/1625 (46.6)	—	34	39.2	26.8	—
Have hobby:						
No	739/1797 (41.1)	<0.001	46.2	40	13.8	<0.001
Yes	1262/4913 (25.7)	—	19	47.3	33.8	—
Frequency of social engagement:						
Less than once a week	191/482 (39.6)	<0.001	38	41.3	20.8	<0.001
1-2 times a week	1175/4100 (28.7)	—	22	47	31	—
≥3 times a week	635/2128 (29.8)	—	31.8	43	25.2	—
Community group engagement:						
Never	1106/3080 (35.9)	<0.001	38.6	43.8	17.6	<0.001
Rarely	566/2170 (26.1)	—	19.8	48.1	32.1	—
Frequently	329/1460 (22.5)	—	9.8	44.5	45.7	—

CES-D=Centre for Epidemiological Studies Depression scale.

P values show results from χ^2^ tests that compare baseline differences. For deaths, percentage is proportion in each category who died during follow-up. For receptive arts engagement, percentage is proportion in each category at baseline.

We stratified analyses by age at which participants’ arts engagement was recorded, whether participants had cancer at baseline, and whether participants had problems that affected mobility. With these adjustments made, the proportionate hazards assumption was met (tested using the Schoenfeld residuals test). To explore the minimum strength of association that any unmeasured confounder would need to fully explain away any association, we calculated the E value, which is a measure of whether the inclusion of further confounders is likely to lead to the attenuation of results.^30^ All analyses were weighted using inverse probability weights to ensure national representation and to take account of differential non-response. We additionally explored whether differences in baseline factors between those who do and do not engage in arts could explain an association between receptive arts engagement and mortality by rerunning analyses using nested models of covariates and by calculating the percentage of protective association explained (PPAE) by including such variables in the model using the equation: PPAE=(HR (E+C+X)–HR (E+C))/(1–HR (E+C))*100, where HR=hazard ratio, E=exposure, C=covariates, and X=explanatory variable being tested.^31^ We confirmed that there were no issues with collinearity and all models met regression assumptions.

We carried out three sets of sensitivity analyses. Our first set assessed whether results were found consistently across subgroups (by rerunning analyses on subgroups) or if certain factors acted as moderators (by including interaction terms in models). In relation to demographics, we tested age and sex specifically. In relation to socioeconomic factors, we tested employment status, wealth, education, and social status. Finally, in relation to social factors, we tested marital status, living alone, loneliness, number of friends, frequency of social engagement, and civic engagement.

Our second set of sensitivity analyses tested with greater rigour whether some of our identified confounders could account for any associations by including a range of further factors that could have acted as confounders. To test whether results were because of physical function, in addition to controlling for sedentary behaviours, we further adjusted for frequency of vigorous physical activity and presence of any mobility problems that affected walking. To test whether broader aspects of socioeconomic status could have confounded associations, we additionally included wealth in tenths rather than fifths for greater nuance; and controlled for retirement status, whether participants were spending time regularly volunteering, or whether participants had undertaken any further education or training in the past 12 months. Further, we considered whether, regardless of socioeconomic status, aspects of life demands, autonomy, or discretion over free time activities could affect ability to engage in cultural activities and longevity. So we additionally adjusted for whether participants felt they had control over what happened in most situations, whether they felt they had different demands in their lives that were hard to combine, and whether they felt they had enough time to do everything they wanted to. Finally, to test whether results were related to accessibility, perhaps because participants lived in more rural areas or areas of higher deprivation, we additionally controlled for the type of residential area in which participants lived (urban *v* town, and fringe *v* village *v* hamlet or other sparse dwelling); we also linked lower layer super output areas data from ELSA with data from the index of multiple deprivation (the official measure of relative deprivation for small areas of England) and included these scores in fifths for each participant in the analysis.

Our third set of sensitivity analyses tested the broader assumptions of our statistical models. To try and reduce the potential for reverse causality (whereby participants had unidentified health conditions that might have affected their participation in cultural activities and predisposed them to premature mortality), we excluded deaths in the two years after baseline. To deal with missing data on covariates and exposure for people who had completed questionnaires but omitted certain items (12%), we used multiple imputation by chained equations using predictor variables in the mortality models to create 20 imputed datasets, which returned the sample size to 7620. We did not impute data on participants who had not completed questionnaires because we could not confirm that these data were missing at random. Additionally, in our main analyses, we used semiparametric methods. But as these did not estimate the baseline hazard, we also tested whether results were consistent when using a parametric model. Because the hazard function showed similarities to an exponential distribution, we used an exponential proportional hazards model, with Akaike’s information criterion and the Bayesian information criterion showing similarity of fit to a Weibull model; however, Wald tests for ĸ=1 confirmed best fit for the exponential model compared with other parametric proportional hazard models tested.

Finally, we used ICD-10 (international classification of diseases, 10th revision) codes to recategorise death by cause of death into four main categories (cardiovascular disease, cancer, respiratory, and other), and reran analyses to determine if results varied by cause of death. Only two participants were omitted owing to unknown cause of death. All analyses were carried out using Stata version 14 (Statacorp).

### Patient and public involvement

In addition to broader patient and public involvement in ELSA (see https://www.ELSA-project.ac.uk), patients and the public were involved in the formulation of this research question through a public engagement event held at University College London in July 2018 that focused on generating new research questions on arts engagement and health outcomes. Patients and the public were not involved in the design or implementation of the study, or in the interpretation or writing up of results. However, plans are in place for their involvement in dissemination of the findings after publication. Dissemination of findings to study participants will be conducted as part of the broader dissemination activity of the ELSA study (https://www.ELSA-project.ac.uk).

## Results

### Demographics

Of the 6710 participants, there were 2001 deaths (29.8%) over the follow-up period. Table 1 shows the demographics of the sample and the relative mortality rate. Men were more likely to die over the follow-up period than women, as were older people, those who were not married or living with a partner, people with no educational qualifications and not currently working, and people of lower wealth and occupational status. Additionally, the mortality rate was higher in people with higher depressive symptoms, with poor eyesight or hearing, with a diagnosis of cancer, lung disease, cardiovascular disease, or other long term condition, in people who were physically inactive, in those who only rarely drank alcohol, and in people who smoked. The rate was also higher in people with lower levels of cognition, in those who were lonely, with no close friends, who lived alone, who did not have a hobby, who only socialised rarely, and who never engaged with community groups.

There was a descriptive dose-response relation across frequency of receptive arts engagement and mortality: 47.5% of people who never engaged in cultural activities died over the follow-up period, but only 26.6% of those who engaged infrequently (less than once a year up to twice a year) and 18.6% of those who engaged frequently (every few months or more). This corresponded to 6.0 deaths per 1000 person years among those who never engaged in cultural activities (95% confidence interval 5.7 to 6.3), 3.5 deaths per 1000 person years among those who engaged infrequently (3.3 to 3.7), and 2.4 deaths per 1000 person years among those who engaged frequently (2.2 to 2.7).****

### Associations with mortality

**Fig 2:**
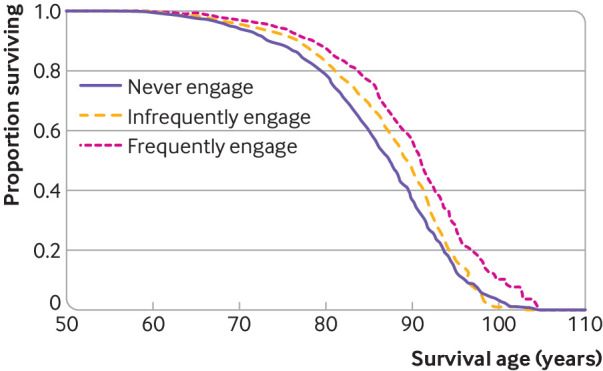
Survivor function, which shows survival age by frequency of receptive arts engagement when adjusting for demographic, socioeconomic, health related, behavioural, and social confounding factors

When we adjusted for age only, cultural engagement (as a continuous variable) was associated with a 33% lower hazard of dying over the follow-up period (hazard ratio 0.67, 95% confidence interval 0.63 to 0.71). This was reduced to a 20% lower hazard when we adjusted for all identified demographic, socioeconomic, health related, behavioural, and social factors (0.80, 0.75 to 0.87). When we considered frequency of cultural engagement in more detail, people who engaged with cultural activities on an infrequent basis (once or twice a year) had a 14% lower risk of dying at any time during the follow-up period (0.86, 0.77 to 0.96) than those who never engaged when we accounted for all confounding factors. Similarly, those who engaged on a frequent basis (every few months or more) had a 31% lower risk of dying at any point during the follow-up period when we accounted for all confounding factors (0.69, 0.59 to 0.80; see fig 2). The E value was 1.91 (lower 95% confidence interval 1.61), which suggests that considerable unmeasured confounding would be needed to explain away the effect estimate.

### Explanatory factors

To consider why the association between arts engagement and mortality exists, we performed staged analyses that explored the percentage of protective association explained. These analyses showed that 41.9% of the association was explained by identified factors. Of these factors, baseline differences in cognition (15.2%), social and civic engagement (12.1%), mobility and disability (12.1%), wealth (9.1%), health behaviours (9.1%), sedentary behaviours (6.1%), and loneliness, living status, and marital status (6.1%) explained the largest proportion of the association. Health conditions and sensory impairment had no discernible impact on the association (table 2).

**Table 2:** Cox proportional hazards models showing associations between receptive arts engagement and 14 year mortality by calculating the percentage of protective association explained by specific confounding factors

Explanatory factors	Adjusted hazard ratio (95% CI)	P	PPAE (%)
Basic model (age)	0.67 (0.63 to 0.71)	<0.001	—
+sex	0.67 (0.63 to 0.72)	<0.001	0
+education, occupational status, and employment status	0.67 (0.63 to 0.72)	<0.001	0
+wealth	0.70 (0.65 to 0.75)	<0.001	9.1
+cancer, lung disease, cardiovascular disease, or other long term condition	0.67 (0.62 to 0.71)	<0.001	0
+mobility and disability	0.71 (0.66 to 0.75)	<0.001	12.1
+depressive symptoms and psychiatric conditions	0.68 (0.64 to 0.72)	<0.001	3.0
+cognition	0.72 (0.67 to 0.76)	<0.001	15.2
+sensory impairment (hearing and eyesight)	0.67 (0.63 to 0.72)	<0.001	0
+sedentary behaviours	0.69 (0.65 to 0.74)	<0.001	6.1
+other health behaviours (drinking and smoking)	0.70 (0.65 to 0.74)	<0.001	9.1
+loneliness, living status, and marital status	0.69 (0.64 to 0.73)	<0.001	6.1
+social, civic, and hobby engagement	0.71 (0.67 to 0.76)	<0.001	12.1
=all	0.80 (0.75 to 0.87)	<0.001	41.9

PPAE=percentage of protective association explained.

Analysed using receptive arts engagement as a continuous variable. Each line of the table shows an explanatory factor or set of explanatory factors added to the basic model. The final line shows all of these factors entered simultaneously.

### Sensitivity analyses

We found no evidence that sex was a moderator, and subgroup analyses by sex showed equivalent results for men and women (supplementary table 1). There was some evidence of a moderating effect of age. Subgroup analyses confirmed associations existed in those whose arts engagement was measured at age 65 and older, but not in those who were younger than 65 (supplementary table 2). We found no evidence of moderating effects of employment status, wealth, or education. Some evidence existed of moderation by social status, but subgroup analyses showed that results were also present among those in managerial and professional occupations, and among those in lower supervisory and semiroutine occupations (supplementary table 3). We found no evidence that social factors including living alone, loneliness, number of friends, or frequency of social contact or civic engagement acted as moderators.

Associations were consistent when we additionally adjusted for any mobility problems with walking and engagement in vigorous physical activity (supplementary table 4). Results did not change when we also adjusted for broader aspects of socioeconomic status, including more nuanced indices of wealth, retirement, volunteering, education, and deprivation (supplementary table 5); or when we considered how in control of their lives people felt, whether they felt they had competing demands to deal with, and whether they felt they had sufficient time available (supplementary table 6). Associations were also consistent when we also adjusted for the type of location in which people lived and the deprivation of that location (supplementary table 7), with no evidence of moderation by these factors.

Results were maintained when we excluded deaths within the first two years after baseline (supplementary table 8). We also found no change in results when we imputed missing data (supplementary table 9), or when we used an exponential model (supplementary table 10). Finally, results did not differ across all four major causes of death (cardiovascular disease, cancer, respiratory, and other; supplementary table 11).

## Discussion

### Principal findings

This study explored whether receptive arts engagement could have protective associations with survival. We analysed the longitudinal relation between receptive arts engagement and mortality across a 14 year follow-up period in a nationally representative sample of adults aged 50 and older. Results showed a dose-response relation: risk of dying at any point during the follow-up period among people who engaged with cultural activities on an infrequent basis (once or twice a year) was 14% lower than in those with no engagement; for those who engaged on a frequent basis (every few months or more), the risk was 31% lower. The association was independent of all identified confounders, was found across all major causes of death, and was robust to a wide range of sensitivity analyses.

### Strengths and limitations of study

This study had several strengths. We used a nationally representative sample of older adults, applied data linkage to national mortality data, and included a comprehensive list of identified confounders. However, several limitations exist. Firstly, this study was observational, and although we took a number of additional steps to try and test the assumptions of models, causality cannot be assumed. It remains possible that unidentified confounding factors could account for the associations found. However, our E value suggests that such unmeasured confounders would need to have a considerable effect to account for the associations found. Secondly, most of our data were based on participant self report (eg, existing clinical diagnoses of health conditions), so data might be affected by self report bias. However, participants were unaware of the specific hypothesis of this study. Relatedly, there could be measurement error and misclassification (especially for categorical variables) in our confounders that might result in our analyses not being fully adjusted and leaving residual confounding. Thirdly, in our analyses on cause of death, we focused on four major categories, but owing to insufficient power it remains unclear how results differed for specific subsets of cause of death.^22^

### Comparison with other studies

Our results build on previous broad literature on leisure activities and mortality, and more specifically, on the findings from two previous analyses of Scandinavian data.^23^ ^24^ However, our study extends these findings in three key ways. Firstly, the results show the association in another national population. Recognised cross cultural differences exist in the consumption and value of receptive arts engagement, therefore the replication of results in a different country is important because it suggests the association is not confined to one particular cultural context.^32^ ^33^ Secondly, we found no evidence of moderation by sex. Previous research suggests that men and women are affected differently by protective factors. For example, daily reading has been associated with survival in men but not women,^34^ while leisure participation broadly has been found only to be beneficial in men.^19^ However, another previous study found that receptive arts engagement was the only leisure activity that did not appear to show a differential survival association by sex.^35^ Our study supports this finding and showed no moderating effect and similar protective associations for men and women

Thirdly, our study identified some of the potential factors that could act as mechanisms that underpin the protective association with mortality. Part of the association is attributable to differences in socioeconomic status among those who do and do not engage in the arts, which aligns with research that suggests engagement in cultural activities is socially patterned.^36^ ^37^ However, the association remains independent of socioeconomic status, so this does not fully explain the association. Some of the other factors that accounted for part of the association included mental health and cognition. This finding is consistent with research that shows that receptive arts engagement can help in preventing and managing depression, and that it can provide support in preventing cognitive decline and in developing cognitive reserve. Our results are also consistent with research that suggests poor mental health and lower cognition can be barriers to engaging in arts activities.^2^ ^4^ ^38^ Similarly, other social and civic engagement explained some of the association, which ties in with well known literature on social activity and mortality.^11^ However, this study also showed that the association is independent of all of these factors, and over half of the association remains unexplained.

When considering what could explain this remaining association, research has suggested that arts engagement builds social capital, which improves people’s access to knowledge and resources, and could help with successful ageing.^10^ Further possibilities are that arts engagement improves a sense of purpose in life, helps with the regulation of emotions and thereby enhances coping, supports the buffering of stress, and builds creativity, which improves people’s ability to adapt positively to changing life circumstances.^16^ ^17^ ^39^ The potential mediating role of these factors remains to be explored further in future studies.

### Conclusions and future research questions

In conclusion, this study suggests that receptive arts engagement could have independent longitudinal protective associations with longevity in older adults. This association appears to be partly explained by differences in factors such as cognition, mental health, and physical activity among those who do and do not engage in the arts, but seems to be independent of these factors. This study did not compare the relative effect size of arts and other known predictors of mortality, but other factors such as socioeconomic status, physical health, and health behaviours undoubtedly have a larger bearing on mortality risk. 

This study raises a number of future research questions. Firstly, we focused on receptive arts activities but were unable to assess the potential overlap with active participation in arts activities (such as making music, painting, and dancing) because no suitable questions were included in ELSA. So how receptive arts activities compare with active arts activities remains to be explored. Secondly, we assessed receptive arts engagement at a single point in time, but future studies could consider how life trajectories of receptive arts engagement are related to mortality. This study is important because of the current focus on schemes such as “social prescribing” and “community service referrals” that are being used to refer people (including older adults) to community arts activities in a number of countries.^40^ ^41^ ^42^ In addition to other literature that explores the benefits of such engagement for specific mental and physical health conditions, our results suggest that cultural engagement is associated with longevity. A causal relationship cannot be assumed, and unmeasured confounding factors might be responsible for the association.**Nevertheless, our results highlight the importance of continuing to explore new social factors as core determinants of health.

**What is already known on this topic**

There is increasing interest in the health benefits of the arts, and debate about potential evolutionary benefits of arts engagement
Several theories suggest that the arts could support longevity by improving mental health, enhancing social capital, reducing loneliness, developing cognitive reserve, reducing sedentary behaviours, and reducing risk taking behaviours
While “leisure” has been broadly linked to a lower risk of premature death, few studies have focused specifically on arts engagement, and data from the UK are lacking

**What this study adds**

This study followed a nationally representative sample of adults aged 50 and older in England for 14 years and used linked mortality data from National Health Service records
Receptive arts engagement could have a protective association with longevity in older adults
This association could partly be explained by differences in cognition, mental health, and physical activity among those who do and do not engage in the arts, but remains even when the model is adjusted for these factors.

## Notes

Contributors: Both authors designed the study. DF carried out the analyses and drafted the manuscript. Both authors critically appraised the manuscript and approved it for submission. Both authors had full access to the data and can take responsibility for the integrity of the data and the accuracy of the data analysis. Both authors act as guarantors. The corresponding author attests that all listed authors meet authorship criteria and that no others meeting the criteria have been omitted.

Ethical approval: The study received ethical approval from the National Research Ethics Service and all participants provided informed consent.

Data sharing: The data involved in this analysis are available through the UK Data Service.

The lead author (DF) confirms the manuscript is an honest, accurate and transparent account of the study being reported, no important aspects of the study have been omitted and any discrepancies from the study as originally planned have been explained.
